# Associations between domain-specific physical activity, sedentary behavior, and binge drinking among adults in the United States and China: evidence from a cross-sectional study, daily tracking, and randomized controlled trial

**DOI:** 10.3389/fpubh.2025.1704171

**Published:** 2025-11-24

**Authors:** Zhuoxiao Liu, Chiwei Huo, Xiaoling Zhang

**Affiliations:** 1 School of Physical Education, Shanghai University of Sport, Yangpu, Shanghai, China; 2 School of Public Health, Zhengzhou University, Zhengzhou, Henan, China

**Keywords:** binge drinking, work physical activity, transportation physical activity, recreational physical activity, sedentary behavior

## Abstract

**Background:**

Binge drinking is linked to a range of diseases and has become a global public health concern. Engaging in physical activity may help reduce the prevalence of binge drinking and excessive alcohol consumption. However, the association may vary depending on the type of physical activity. This study aimed to investigate the association between different types of moderate-to-vigorous physical activity and binge drinking.

**Materials and methods:**

This study included: (1) a cross-sectional survey included 6,707 individuals aged 20 years and older from the NHANES database between 2015 and 2018 (3,473 men and 3,234 women). Physical activity was assessed using the Global Physical Activity Questionnaire, and binge drinking was assessed using the Alcohol Use Questionnaire (ALQ). Binary logistic regression analysis was performed. (2) Daily tracking surveys collected daily data on the duration of physical activity and alcohol consumption over eight consecutive days from 80 individuals, resulting in 640 valid self-reported data points, which were analyzed using a generalized linear mixed-effects model. (3) A randomized controlled trial included 40 participants, randomly divided into a control group (20 individuals) and an experimental group (20 individuals). The experimental group engaged in 4 weeks (28 days) of group square dance recreational physical activity, while the control group had no assigned tasks. Paired-sample *t*-tests and independent-sample *t*-tests were used for analysis.

**Results:**

A cross-sectional survey revealed work physical activity was positively associated with binge drinking (*p* < 0.001; OR = 1.50; 95%CI: 1.30–1.75). Transportation physical activity was positive associated with binge drinking (*p* = 0.002; OR = 1.30; 95%CI: 1.10–1.53). In contrast, recreational physical activity was negatively associated with binge drinking (*p* < 0.001; OR = 0.68; 95%CI: 0.58–0.79). Daily tracking surveys have externally validated the association between work-related physical activity and alcohol abuse (*β* = 0.002, *p* < 0.001). The association between transportation physical activity and alcohol abuse (*β* = 0.014, *p* < 0.001). A randomized controlled trial strengthened the association between recreational physical activity and alcohol abuse, showing that an association between increased recreational physical activities and reduced alcohol abuse (*p* < 0.001).

**Conclusion:**

Different types of physical activity are associated with different patterns of binge drinking. Therefore, when using physical activity as a measure to control binge drinking, it is necessary to pay attention to the type and duration of physical activity.

## 1 Introduction

The National Institute on Alcohol Abuse and Alcoholism (NIAAA) defines binge drinking as a drinking pattern that results in a blood alcohol concentration (BAC) of 0.08% (or 0.08 grams of alcohol per decilitre) or higher. For the average adult, this corresponds to the consumption of five or more drinks per occasion for men and four or more drinks per day for women (1). In the United States, a ‘standard drink’ is defined as containing 0.6 fluid ounces (14 grams) of pure alcohol. The World Health Organization (WHO) identifies ethanol, the primary ingredient in alcoholic beverages, as psychoactive toxic substance that can lead to dependence and is associated with numerous health risks. Alcohol use contributes to more than 200 diseases, injuries, and health conditions globally. In 2019, alcohol-related causes were responsible for 2.6 million deaths worldwide, including 2 million men and 600,000 women (2). The U. S. Dietary Guidelines advise adults of legal drinking age to either abstain from alcohol or limit their consumption to no more than two drinks per day for men and one for women, as consuming less alcohol is healthier than drinking more (2). Therefore, binge drinking is a significant social concern, and identifying its potential risk factors while providing early prevention and intervention measures is essential.

Recent research has explored the relationship between physical activity and sedentary bahavior and binge drinking, though findings remain inconsistent (3–5). Physical activity is defined as any bodily movement produced by skeletal muscles that results in energy expenditure (6). In contrast, sedentary behavior refers to any waking behavior characterized by an energy expenditure ≤1.5 metabolic equivalents while in a sitting, reclining, or lying posture (7).

Some studies suggesting a positive correlation between physical activity and binge drinking, but they differ in the measurement methods used: many rely on self-report questionnaires (8), which may overestimate or mis-classify activity intensity/duration, while a few use device-based measures like accelerometers or wearable monitors which are more objective but less common (9). These differences in measurement could contribute to inconsistent findings, because questionnaire-based and device-based assessments often yield different estimates of the same behaviors. Furthermore, meta-analytic work (7) has begun to synthesize across studies accounting for measurement types, which could help clarify whether intensity or duration of physical activity truly increases risk of binge drinking (10, 11). This is particularly evident across different populations, occupations, income levels, and educational backgrounds. In specific groups such as adolescents aged 14–16, female university students, male university students, and individuals over 50 years old, higher levels of physical activity are associated with higher alcohol consumption and heavier drinking habits, while lower physical activity levels are linked to lower alcohol consumption and lighter drinking habits (12–15). In different occupational fields, alcohol consumption is more prevalent among members of sports clubs and those in physically demanding occupations. However, some studies suggest (16, 17) that increasing physical activity can help reduce binge drinking, with moderate-intensity exercise being most effective in controlling binge drinking. This is particularly evident in various forms of exercise (walking, running, resistance training, yoga, etc.), where alcohol consumption significantly decreases (18).

There are differing perspectives on sedentary behavior as a distinct behavioral pattern in relation to binge drinking, specifically concerning variations in sedentary duration and measurement methodologies. Some studies indicate no significant association between sedentary behavior and drinking (19, 20), while another study found that as sedentary behavior time increases, the probability of drinking decreases, which contradicts the conclusions of some studies suggesting a positive correlation between sedentary behavior and drinking (9). The inconsistency in findings regarding sedentary behavior may depend on variations in measurement methods. Many of them use self-report instruments (21) (e.g., sitting time questionnaires, logs, surveys) that ask participants to recall hours spent sitting in various domains (e.g., work, leisure, transport). Other studies incorporate device-based measures such as accelerometers or inclinometers (22), which capture movement or posture continuously over a period of days.

It is necessary to adopt a complementary framework to understand the inconsistent empirical associations between physical activity and alcohol abuse. The stress coping model proposes that both exercise and alcohol may function as coping strategies—exercise often as an adaptive response and alcohol as a maladaptive one—so individuals’ relative reliance on different coping styles will shape whether physical activity substitutes for or co-occurs with drinking (23). The compensation hypothesis predicts the opposite dynamic: people may feel licensed to drink more after engaging in health-promoting behaviors (e.g., “I exercised today, so I can drink”), which would produce a positive association between activity and alcohol use (24). A self regulation perspective adds a resource-based mechanism: because self-control is finite, when individuals exhaust their self-control resources during exercise or other energy-demanding activities, they may subsequently be more prone to impulsive drinking behavior (25). Neural reward mechanism emphasizes that both exercise and alcohol engage brain reward systems; thus, shared neurobiological reinforcement may help explain why some individuals pursue both behaviors, or why exercise can sometimes heighten reward sensitivity to substances (26). Finally, the theory of common liability posits that the relationship between physical activity and alcohol consumption may stem from both being influenced by common personality traits or social environments (such as a drive for novelty, social factors, or genetic predispositions), rather than one behavior directly causing the other (27).

These frameworks exhibit some conceptual overlap, yet they highlight distinct operational mechanisms necessitating different empirical research approaches. Specifically, perspectives on stress coping and self-regulation emphasize situational factors such as perceived stress and self-control depletion, suggesting that associations between physical activity and alcohol consumption may vary depending on context or timing. The compensation hypothesis focuses on internalized license effects, whereby individuals may perceive justification for drinking after engaging in health-promoting behaviors such as exercise. In contrast, the reward overlap perspective highlights that both physical activity and alcohol activate shared dopamine reward pathways. Finally, the shared liability framework attributes the observed association to stable trait or environmental confounders that collectively predispose individuals toward concurrent engagement in both behaviors. Integrating these frameworks motivates our decision to disaggregate physical activity by purpose (work, transport, recreation) and to examine both between-person and within-person (daily) associations, since different activity types and contexts are likely to map onto different mechanisms (coping vs. compensation vs. shared vulnerability).

In summary, previous studies have shown that while physical activity can influence the likelihood of binge drinking, not all forms of physical activity have a negative impact on binge drinking. Many studies have indicated that the intensity, duration, and type of physical activity can have different effects on binge drinking (4, 10, 11). Based on the above review of relevant literature, although there is a correlation, the research results are inconsistent. We speculate that the differing purposes and motivations for engaging in physical activity may account for these discrepancies. Therefore, this study categorizes physical activity into different types based on the participants’ purposes for engaging in physical activities, including work-related physical activity, transportation physical activity, and recreational physical activity. In addition, sedentary behavior is examined as a distinct but related behavioral domain. This approach aims to further explore the association between physical activity and binge drinking. Given regional differences in behavior patterns around the world, it is unclear whether different types of physical activity, which are influenced by environmental, political, economic, cultural and institutional factors, will show consistency with alcohol abuse in the United States. Therefore, this study assessed and verified the association between different types of physical activity and sedentary behavior and alcohol abuse through cross-sectional surveys, daily tracking surveys and randomized controlled trials.

## 2 Materials and methods

This study comprised three complementary studies designed to examine the association between different types of physical activity and binge drinking among adults.

Study 1, a cross-sectional analysis based on 6,707 adults from the 2015–2018 NHANES database, investigated population-level associations between work, transportation, and recreational physical activity and binge drinking.

Study 2, a daily tracking survey of 80 participants, provided short-term longitudinal validation of the relationships between work physical activity and transportation physical activity and alcohol consumption in real-life contexts.

Study 3, a randomized controlled trial (RCT) involving 40 participants, experimentally tested the association of recreational physical activity on reducing binge drinking.

Together, these three studies form a multi-level research framework combining population-based, daily behavioral, and experimental evidence.

### 2.1 Study 1: Cross-sectional study

#### 2.1.1 Participants and procedures

**Figure 1:**
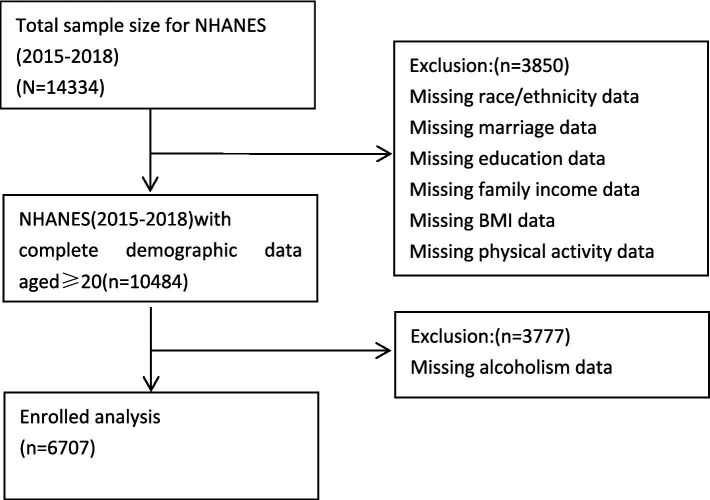
Flow chart of subject selection.

The National Health and Nutrition Examination Survey (NHANES) is a population-based cross-sectional survey designed to collect information on the health and nutritional status of adults in the United States (10). The data used in this study were obtained from a representative sample of the 2015–2018 NHANES survey, which included 6,707 adults aged 20 years and older. All members included in the NHANES survey cycle had resided in the United States for 2 months or longer. NHANES has been approved by the National Centre for Health Statistics Research Ethics Review Committee; all adult participants provided written informed consent (28). Detailed data and informed consent information are available on the NHANES official website.^1^ For more detailed information on study design, sampling, and exclusion criteria, see the figure below (see Figure 1).

#### 2.1.2 Measurement

##### 2.1.2.1 Physical activity assessment

The physical activity data were extracted from the NHANES database. Assessment was conducted using a physical activity questionnaire, which provided interview-based data regarding the participants’ levels of physical activity. These domains were pre-defined in the NHANES dataset and were not recategorized by the authors (29). Physical activity data were obtained from the Physical Activity Questionnaire (PAQ) in NHANES, which was developed based on the World Health Organization’s Global Physical Activity Questionnaire (GPAQ). The PAQ includes questions PAQ605–PAQ680 and collects respondent-level information on work, transportation, and recreational physical activities (30). The questionnaire provided interview data on participants’ physical activity levels. Based on the established classification in physical activity studies (31, 32), we categorized individuals engaged in moderate-to-vigorous intensity activities into two groups: ‘moderate-to-vigorous intensity work physical activity’ and ‘moderate-to-vigorous intensity recreational physical activity.’ Additionally, transportation activities such as walking and cycling were classified as transportation physical activity. For the purpose of data analysis, and following the classification approach adopted in (32), participants who reported ≥420 min of sedentary time per day were categorized as having sedentary behavior, whereas those reporting <420 min were classified as non-sedentary. The codes ‘0’ and ‘1’ were used in the final database to represent non-sedentary (<420 min) and sedentary behavior (≥420 min).

##### 2.1.2.2 Binge drinking assessment

The binge drinking database is derived from NHANES, using self-reported data provided by participants and assessed via the Alcohol Use Questionnaire (ALQ). This binge drinking dataset provides information on drinking habits, alcohol consumption, and alcohol use classification. Participants aged 18 and older were eligible for inclusion. These questions were asked by trained interviewers using a computer-assisted personal interview (CAPI) system in the participants’ homes. The ALQ is part of the standardized NHANES alcohol use module, which has been used continuously in national surveillance since 1999. Its items and procedures have demonstrated acceptable validity and reliability in assessing self-reported alcohol consumption (33). Therefore, the ALQ is considered a valid and reliable instrument for large-scale epidemiological studies. Based on the NIAAA’s definition of binge drinking: drinking 4–5 or more drinks per day (1) and recommendations for updating the database questions, we analyzed ALQ question 151, which asks participants, ‘Do you drink 4–5 or more drinks per day?’ In the final database, the codes ‘0’ and ‘1’ represent non-alcoholics and alcoholics, respectively.

##### 2.1.2.3 Covariates

The covariates in this study included demographic data (34, 35) (age, gender, race, education level, marital status), income poverty comparison (36), body mass index (BMI), and health status (37). A total of 6,707 participants were divided into five racial groups: Mexican American, Hispanic, non-Hispanic white, non-Hispanic black, and other races. Education level was divided into no high school completion, high school graduation, and post-high school. Marital status was categorized as cohabiting (married, living with a partner), married but living alone (widowed, divorced, or separated), and never married. The income poverty ratio was calculated by dividing household income by the poverty line standard for the survey year. In this study, income and poverty rates were used to create two income status categories: poverty (<1.3) and middle income (≥1.3) (38). BMI is categorized as underweight (<19 kg/m^2^), normal (19–24.9 kg/m^2^), overweight (25–29.9 kg/m^2^), and obese (≥30 kg/m^2^) (39). Health status is categorized into five levels: excellent, very good, good, fair, and poor (39).

#### 2.1.3 Statistical analysis

We used Microsoft Excel 2010 to extract and summarize the raw data, excluding options such as ‘refused,’ ‘do not know,’ and ‘missing.’ All analyses followed the National Health and Nutrition Examination Survey (NHANES) analytical guidelines. Four-year MEC examination weights (WTMEC4YR) were applied for the 2015–2018 cycle to obtain nationally representative estimates. Variance estimation incorporated the stratification (SDMVSTRA) and clustering (SDMVPSU) design variables. Weighted logistic regression models were used to estimate odds ratios (ORs) and 95% confidence intervals (CIs).

Descriptive statistics and chi-square tests were first used to assess the significance of demographic information, different types of physical activity, and sedentary behavior in relation to alcohol abuse. A binary logistic regression model was then employed to evaluate the independent association between moderate-to-vigorous intensity work-related physical activity, transportation-related physical activity, leisure-time physical activity, and sedentary behavior with alcohol abuse. All data were analyzed using the social science statistical software package SPSS version 29.0. *p*-values less than 0.05 were considered statistically significant (two-tailed test). Variables that were statistically significant in univariate analysis were included in stepwise binary logistic regression analysis.

In binary logistic regression analysis, we used moderate-to-vigorous physical activity related to work, transportation, and leisure as independent variables (0 = no, 1 = yes), or sedentary behavior as an independent variable (0 < 420 min, 1 ≥ 420 min). Binge drinking (0 = non-binge drinking, 1 = binge drinking) was used as the dependent variable. In each individual regression analysis of the independent variables, three models were derived: one without excluding covariates, one excluding demographic factors, and one excluding all covariates for testing.

To explore the potential nonlinear association between different types of physical activity and alcohol abuse (40, 41), we used R 4.5.1 software to generate restricted cubic spline curves (RCS), primarily to assess the dose–response relationship between different types of physical activity duration. Following Harrell’s recommendation, the nodes are positioned at the 5th, 35th, 65th, and 95th percentiles of the physical activity duration distribution.

### 2.2 Study 2: Daily tracking survey

#### 2.2.1 Participants and process

The data for this study was collected in Jinan City, Shandong Province, China. In July 2025, eligible heavy drinkers were recruited through online recruitment advertisements. Participants completed an online diary questionnaire (demographic and baseline data). All participants provided written informed consent. A total of 83 heavy drinkers agreed to participate in the study. Data on the duration of work physical activity, transportation physical activity, and daily alcohol consumption were collected over eight consecutive evenings. Each evening between 22:00 and 24:00, participants self-recorded and reported their daily work physical activity duration, transportation physical activity duration, and alcohol consumption. These reports were cross-checked with the pedometer-based activity data to ensure consistency. Three participants were excluded due to unexplained absences or physical discomfort resulting in insufficient data, leaving a total of 80 participants with 640 daily reports (42 males, average age 29.84, standard deviation 7.134, age range 19–45 years). The participant attrition rate was 4%, the number of valid reporting days was 8, and the response rate was 97.5%.

#### 2.2.2 Measurement

Participants were instructed to use their own wearable devices with pedometer and physical activity monitoring functions (e.g., Huawei Band, Xiaomi Mi Band, Apple Watch, Fitbit, and Garmin). Since the study relied on participants’ existing personal devices, no single standardized brand was specified. During the survey, participants reported that most of their devices were capable of continuously recording daily step counts, duration of moderate-to-vigorous physical activity, and exercise sessions.

##### 2.2.2.1 Duration of physical activity at work

Participants should follow the definition of work physical activity (first, think about the time you spend working. Consider work as something you must do, such as paid or unpaid work, household chores, and yard work. Does your work involve moderate to high-intensity activities that increase breathing or heart rate, such as continuous lifting or carrying heavy objects, digging, or construction work for at least 10 min?), and combine self-reported data on the duration of work physical activity for the day using smart bands, wrist-worn pedometers, and smartwatches.

To avoid mixing the duration of transportation physical activity and recreational physical activity in the report. Transportation physical activity duration (minutes) is calculated based on step count, and participants remove smart bracelets or similar devices during recreational physical activities. Participants were instructed to remove smart devices during recreational physical activities to prevent misclassification of activity types, as accelerometer-based devices cannot distinguish between transportation and recreational physical activity contexts. Although this procedure minimized classification bias, it may have led to a slight underestimation of total recreational physical activity.

Work physical activity duration equals total exercise duration minus transportation physical activity duration. Duration is reported in minutes. We retain work physical activity duration as quantitative data for subsequent statistical analysis.

##### 2.2.2.2 Duration of transportation physical activity

Participants self-reported the duration of their transportation physical activity for the day based on the definition of transportation physical activity (the usual way you travel to and from places such as school, shopping, and work. Do you walk or cycle continuously for at least 10 min to and from certain places?). The duration was reported in minutes. We retained the duration of transportation physical activity as quantitative data for subsequent statistical analysis.

##### 2.2.2.3 Alcohol consumption

Based on the NIAAA’s definition of alcohol abuse and the CDC’s drinking standards, we have approximated the concentration and volume of various common alcoholic beverages, with the corresponding ‘standard drink’ shown below (see Table 1). Participants recorded and reported the number of drinks consumed per day based on the type of alcohol they drank.

**Table 1:** Standard cup sizes for common alcoholic beverages.

Variable	Common alcohol concentrations (ABV)	Approximate capacity (ml)
Regular beer	≈5%	350 mL
Table wine	≈12%	150 mL
Spirits	≈40%	44 mL

#### 2.2.3 Statistical analysis

We used Spearman’s correlation analysis and generalized linear mixed models (negative binomial regression) to examine the association between individual work physical activity, transportation physical activity, and alcohol consumption.

The correlation analysis was conducted for each participant, calculating the average daily duration of work physical activity, transportation physical activity, and average daily alcohol consumption over an 8-day observation period to reduce the impact of individual daily fluctuations and more accurately reflect their daily behavior patterns. We then calculated the Spearman correlation coefficient (*ρ*) and corresponding *p*-values to preliminarily assess the direction and strength of the association between the two variables.

Subsequently, a multilevel negative binomial regression model was fitted, treating daily drinking occasions (Level 1) as nested within participants (Level 2). The dependent variable was the number of alcoholic drinks consumed per recording, and the main independent variable was the duration of transportation-related physical activity. A random intercept was specified for each participant to account for between-person variability in baseline drinking frequency, while fixed effects estimated within-person associations between physical activity and alcohol consumption. Model parameters were estimated using restricted maximum likelihood (ML).

All analyses were conducted in R version 4.5.1 software, using the lme4 package for model fitting, with a significance level set at *α* = 0.05.

### 2.3 Study 3: Randomized controlled trial

Unlike recreational physical activity, transportation and work physical activity are highly embedded in individuals’ daily routines and are influenced by contextual constraints (commuting distance, job requirements, work schedule). These forms of activity are difficult to manipulate experimentally in a randomized controlled trial, since imposing artificial changes may disrupt participants’ work responsibilities or commuting habits and raise ethical and practical concerns. Therefore, we adopted an intensive longitudinal design (daily tracking survey) to capture natural variations in transportation and work activity and their associations with alcohol consumption.

#### 2.3.1 Participants and procedures

Eligible subjects were recruited through community notice boards and the distribution of flyers. This study adopted a convenience sampling approach, as participants were volunteers recruited from local communities rather than randomly selected from a population database. Four participants were excluded due to loss to follow-up, resulting in a final sample of 40 individuals (28 males; mean age 39.4 ± 9.68 years, range 23–61 years).

Inclusion criteria included: (1) age over 18 years; (2) self-reported good physical and mental health; (3) absence of musculoskeletal or cardiovascular conditions that could restrict participation in physical activity; (4) daily consumption reaching or approaching the criteria for binge drinking; and (5) willingness to participate throughout the entire intervention period.

Exclusion criteria included: (1) binge drinking below standard (<4 cup/day); (2) history of severe cardiovascular, neurological, or psychiatric disorders; (3) ongoing use of medications that might affect physical performance or mood; (4) recent injuries or surgery limiting physical activity; and (5) failure to provide informed consent or incomplete baseline data.

Prior to the trial, all participants signed informed consent forms and were randomly assigned to two groups by drawing envelopes: 20 participants in the experimental group and 20 in the control group. Participants in the experimental group were informed that the intervention involved group square dancing as a recreational physical activity, which took place in a community in Shizhong District, Jinan City, Shandong Province, China, and lasted for 4 weeks (28 days).

To ensure the fairness and validity of the trial, a computer-generated random sequence was used, and allocation concealment was maintained through sealed, opaque, and sequentially numbered envelopes. The researchers responsible for participant allocation were highly experienced in conducting clinical trials; however, they were blinded to the study hypotheses and had no authority to alter or influence the allocation process, thereby minimizing selection bias.

In order to minimize response bias and enhance the validity of the results, participants were kept unaware of the specific objectives and hypotheses of the study throughout the experiment. We only informed them that the study aimed to investigate the general relationship between adults’ lifestyles and health status, without disclosing our focus on the association between specific variables. This approach avoids participants intentionally adjusting their behavior or self-reported data due to knowledge of the study’s focus, thereby reducing directional bias in the results.

#### 2.3.2 Measurement

##### 2.3.2.1 Alcohol consumption

Based on the NIAAA’s definition of alcohol abuse and the CDC’s drinking standards, we have approximated the concentration and volume of various common alcoholic beverages, with the corresponding ‘standard drink’ shown above (see Table 1). Participants recorded and reported the number of drinks consumed each day based on the type of alcohol they drank, with the calculation period for each day being 00:00–24:00.

#### 2.3.3 Statistical analysis

We used SPSS 29 statistical software to process the collected data, performed descriptive statistics on demographic information, and conducted intra-group and inter-group consistency tests on alcohol consumption. We used an independent samples *t*-test to analyze the relationship between recreational physical activity and alcohol consumption in the experimental group and the control group.

To visually illustrate the between-group differences and variability in recreational physical activity and alcohol consumption, we used SPSS 29.0 to generate error chart. Error chart present the central tendency and uncertainty range of each group in the form of means and their 95% confidence intervals (95% CI), aiding in the comparison of differences between the experimental and control groups in the target indicators. In conjunction with the corresponding statistical test results, error chart in this study are primarily used to visualize the direction, magnitude, and statistical significance of intergroup differences.

## 3 Results

### 3.1 Study 1: Cross-sectional study

#### 3.1.1 Demographic characteristics

This study included a total of 6,707 adults aged 20 years or older, with 51.78% being male and 48.22% being female. They participated in the 2015–2018 NHANES survey and completed data on physical activity, alcohol consumption, and other demographic information. There were significant differences between the heavy drinking group and the non-heavy drinking group in terms of age, gender, race, education level, marital status, poverty ratio, health status, work physical activity, transportation physical activity, and recreational physical activity. No differences were observed in body mass index and sedentary behavior (see Table 2).

**Table 2:** Demographic characteristics of adults aged ≥20 years classified by alcohol consumption in the NHANES 2015–2018.

Characteristics, *n*%	Sample capacity	Binge drinking	Do not binge drinking	Statistic	*P*
	Total (*n* = 6,707)	0 (*n* = 5,670)	1 (*n* = 1,037)		
Age, M (Q₁, Q₃)	49.00 (34.00, 63.00)	48.00 (33.00, 63.00)	53.00 (39.00, 64.00)	*Z* = −5.72	<0.001***
Gender				*χ*^2^ = 233.39	<0.001***
Male	3,473 (51.78)	2,710 (47.80)	763 (73.58)		
Female	3,234 (48.22)	2,960 (52.20)	274 (26.42)		
Race				*χ*^2^ = 35.91	<0.001***
Mexican American	949 (14.15)	817 (14.41)	132 (12.73)		
Other Hispanic	669 (9.97)	572 (10.09)	97 (9.35)		
Non-Hispanic White	2,618 (39.03)	2,147 (37.87)	471 (45.42)		
Non-Hispanic Black	1,461 (21.78)	1,228 (21.66)	233 (22.47)		
Other Race	1,010 (15.06)	906 (15.98)	104 (10.03)		
Education				*χ*^2^ = 92.51	<0.001***
Below high school	1,108 (16.52)	856 (15.10)	252 (24.30)		
High school graduate	1,585 (23.63)	1,287 (22.70)	298 (28.74)		
Post high school	4,014 (59.85)	3,527 (62.20)	487 (46.96)		
Marital statues				*χ*^2^ = 16.60	<0.001***
Cohabitation	4,045 (60.31)	3,458 (60.99)	587 (56.61)		
Married living alone	1,427 (21.28)	1,157 (20.41)	270 (26.04)		
Never married	1,235 (18.41)	1,055 (18.61)	180 (17.36)		
Income to poverty				*χ*^2^ = 78.57	<0.001***
Impoverished	1808 (26.96)	1,412 (24.90)	396 (38.19)		
Moderate income	4,899 (73.04)	4,258 (75.10)	641 (61.81)		
BMI				*χ*^2^ = 5.47	0.141
Underweight	141 (2.10)	118 (2.08)	23 (2.22)		
Normal weight	1,627 (24.26)	1,369 (24.14)	258 (24.88)		
Overweight	2,126 (31.70)	1829 (32.26)	297 (28.64)		
Obese	2,813 (41.94)	2,354 (41.52)	459 (44.26)		
Healthy				*χ*^2^ = 112.80	<0.001***
Excellent	581 (8.66)	514 (9.07)	67 (6.46)		
Very good	1751 (26.11)	1,565 (27.60)	186 (17.94)		
Good	2,804 (41.81)	2,374 (41.87)	430 (41.47)		
Fair	1,368 (20.40)	1,078 (19.01)	290 (27.97)		
Poor	203 (3.03)	139 (2.45)	64 (6.17)		
Work physical activity				*χ*^2^ = 42.75	<0.001***
Yes	3,465 (51.66)	3,026 (53.37)	439 (42.33)		
No	3,242 (48.34)	2,644 (46.63)	598 (57.67)		
Transportation physical activity				*χ*^2^ = 12.09	<0.001***
Yes	5,212 (77.71)	4,449 (78.47)	763 (73.58)		
No	1,495 (22.29)	1,221 (21.53)	274 (26.42)		
Recreational physical activity				*χ*^2^ = 67.01	<0.001***
Yes	3,291 (49.07)	2,661 (46.93)	630 (60.75)		
No	3,416 (50.93)	3,009 (53.07)	407 (39.25)		
Sedentary behavior				*χ*^2^ = 2.99	0.084
<420 min	4,258 (63.49)	3,575 (63.05)	683 (65.86)		
≥420 min	2,449 (36.51)	2095 (36.95)	354 (34.14)		

χ^2^: Chi-square test, Z: Wilcoxon test *p < 0.05, **p < 0.01, ***P < 0.001. Figures in parentheses represent percentages.

#### 3.1.2 Association between work physical activity and binge drinking

In this logistic regression analysis, the model table presents three sets of results. Model I (not adjust all covariates, named age, gender, race, education level, marital status, income poverty comparison, BMI, and health status) shows that the odds ratio (OR) for the association between moderate-vigorous work physical activity and binge drinking is 1.559 (95% CI: 1.36–1.78); Model II (adjust all covariates) shows an OR = 1.620 (95% CI: 1.30–1.75). The study results indicate that moderate to vigorous work physical activity remains a risk factor for binge drinking, regardless of whether all covariates are adjusted for. Work physical activity is positively associated with an increased risk of binge drinking (see Table 3).

**Table 3:** Logistic regression analysis results for work physical activity and binge drinking.

Mode	*b*	SE	Wald	*P*	OR (95%CI)
I	0.444	0.068	42.322	<0.001***	1.559 (1.364 ~ 1.782)
II	0.483	0.074	42.407	<0.001***	1.620 (1.401 ~ 1.874)

I: The original model without adjustment for any variables. II: Adjustment all covariates. *p < 0.05, **p < 0.01, ***p < 0.001.

**Figure 2:**
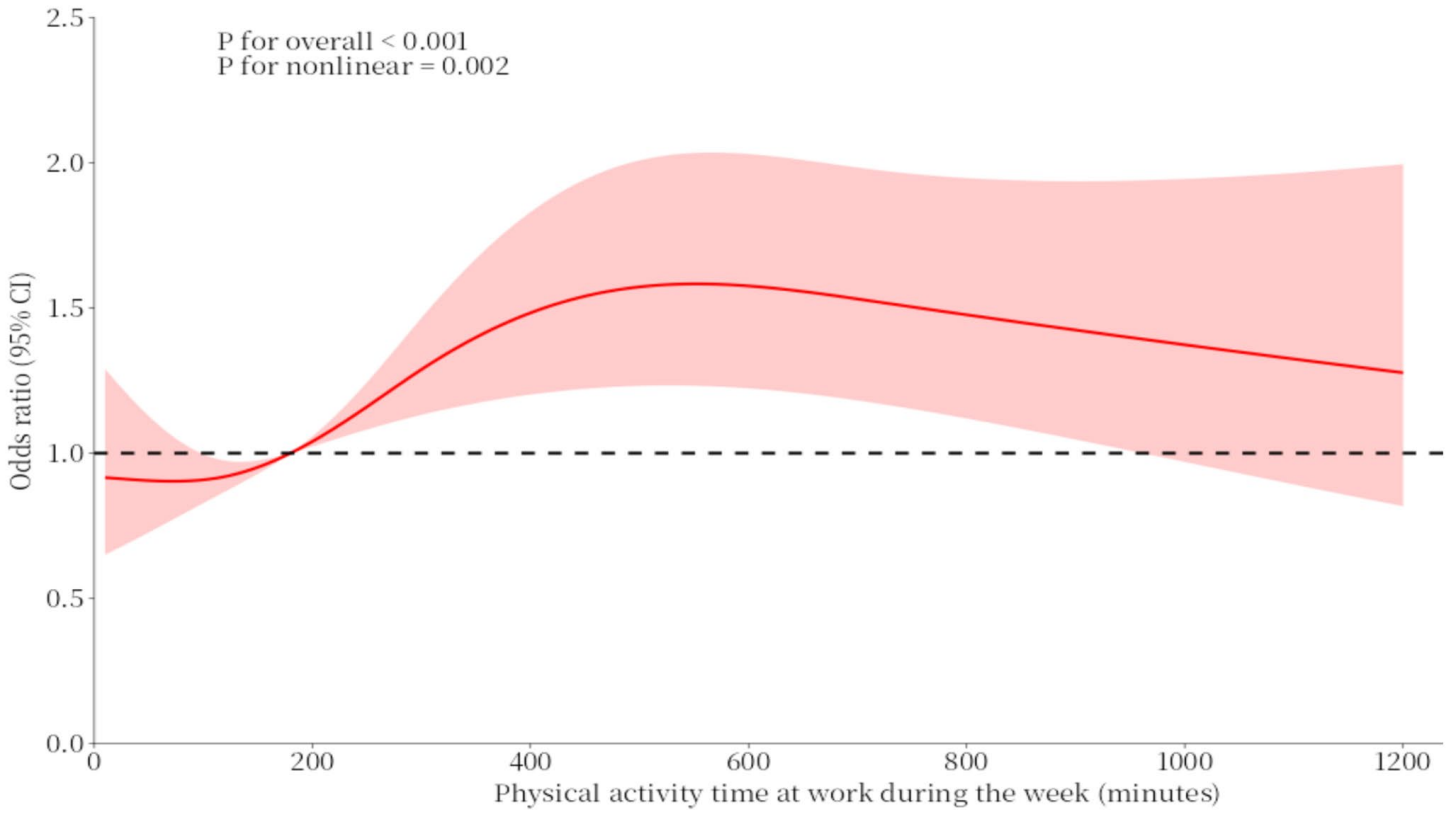
RCS curves of the association between physical activity time at work during the week and binge drinking.

When the duration of daily work physical activity is less than 200 min, the OR for work physical activity and binge drinking is less than 1. When the duration exceeds 200 min, the OR begins to exceed 1. Additionally, when the duration of work physical activity exceeds 550 min, the OR shows a downward trend (see Figure 2).

#### 3.1.3 Association between transportation physical activity and binge drinking

In the logistic regression analysis, Model III (not adjust all covariates, named age, gender, race, education level, marital status, income poverty comparison, BMI, and health status) showed that the OR value for the relationship between transportation physical activity and the risk of binge drinking was 1.308 (95% CI: 1.12–1.52); Model IV (adjust all covariates) showed an OR = 1.258 (95% CI: 1.10–1.53). The study results indicate that, regardless of whether all covariates are adjusted for, transportation physical activity remains a risk factor for binge drinking. Transportation physical activity is positively correlated with an increased risk of binge drinking (see Table 4).

**Table 4:** Logistic regression analysis results for physical activity related to transportation and binge drinking.

Mode	*b*	SE	Wald	*P*	OR (95%CI)
III	0.269	0.077	12.041	<0.001***	1.308 (1.124 ~ 1.523)
IV	0.229	0.084	7.483	0.006**	1.258 (1.067 ~ 1.482)

III: The original model without adjusting for any variables. IV: Adjustment all covariates. *p < 0.05, **p < 0.01, ***p < 0.001.

**Figure 3:**
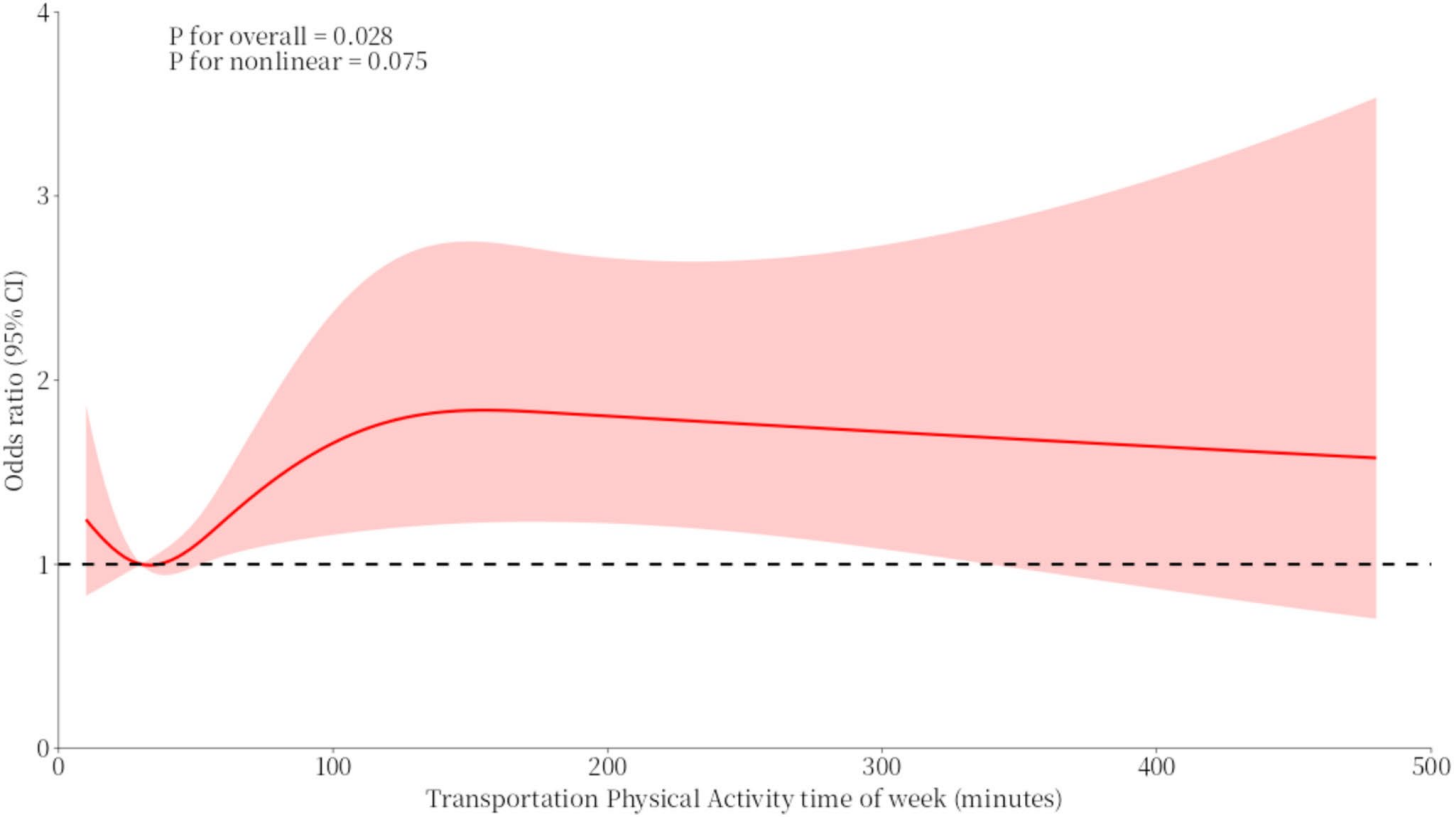
RCS curves of the association between transportation physical activity time of the week and binge drinking.

In the physical activity curve for transportation, the OR values for duration and heavy drinking were both greater than or equal to 1. When the duration was less than 35 min or greater than 145 min, the OR showed a downward trend. When the duration was between 35 and 145 min, the OR showed an upward trend (see Figure 3).

#### 3.1.4 Association between recreational physical activity and binge drinking

In the logistic regression analysis, Model V (not adjust all covariates, named age, gender, race, education level, marital status, income poverty comparison, BMI, and health status) showed that the odds ratio (OR) for the association between moderate to vigorous recreational physical activity and binge drinking was 0.571 (95% CI: 0.50–0.65); Model VI (adjust all covariates) showed an OR = 0.664 (95% CI: 0.58–0.79). The study results indicate that moderate to vigorous recreational physical activity is a protective factor against binge drinking, regardless of whether all covariates are adjusted for. Recreational physical activity is positively associated with a reduced risk of binge drinking (see Table 5).

**Table 5:** Logistic regression analysis results for recreational physical activity and binge drinking.

Mode	*b*	SE	Wald	*P*	OR (95%CI)
V	−0.560	0.069	65.943	<0.001***	0.571 (0.499 ~ 0.654)
VI	−0.049	0.076	28.667	<0.001***	0.664 (0.572 ~ 0.771)

V: The original model without adjustment for any variables. VI: Adjustment all covariates. *p < 0.05, **p < 0.01, ***p < 0.001.

**Figure 4:**
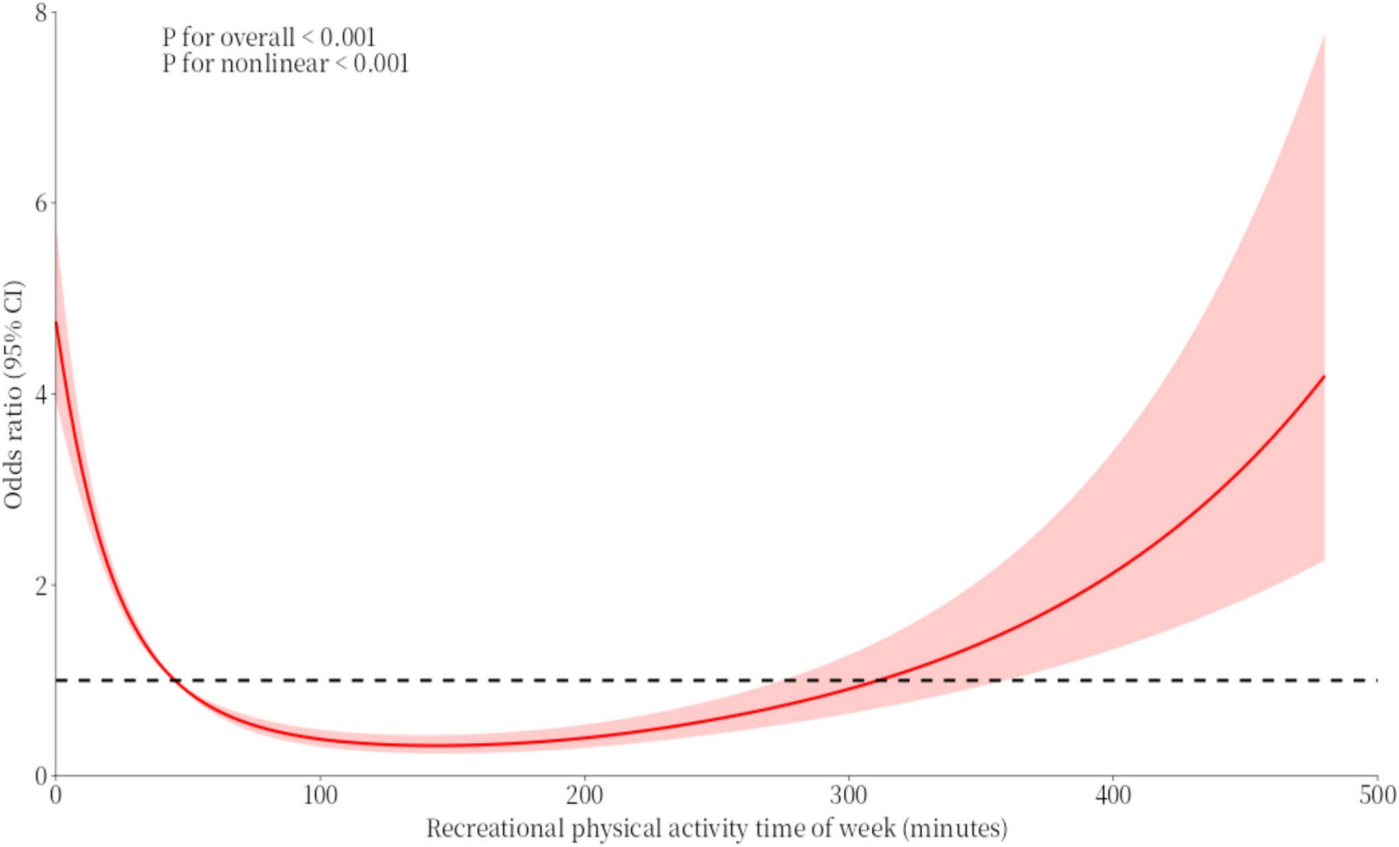
RCS curves of the association between recreational physical activity time of week and binge drinking.

The RCS curve for recreational physical activity and binge drinking generally follows a ‘U’-shaped trend. Before 50 min, the OR value associated with recreational physical activity duration and binge drinking was greater than 1. As recreational physical activity began, the trend of binge drinking decreased rapidly. Between 50 and 300 min, the OR value associated with recreational physical activity duration and binge drinking was less than 1, reaching a low point around 140 min. However, after 300 min, the OR value was greater than 1 and increased rapidly (see Figure 4).

#### 3.1.5 Association between sedentary behavior and binge drinking

In this logistic regression analysis, Model VII (not adjust all covariates, named age, gender, race, education level, marital status, income poverty comparison, BMI, and health status) showed that the OR value for sedentary behavior and the risk of binge drinking was 0.884 (95% CI: 0.77–1.02); Model VII (adjust all covariates) showed an OR = 1.051 (95% CI: 0.85–1.16). The study results indicate that, regardless of whether covariates are adjusted for, sedentary behavior does not significantly differ from the probability of binge drinking. Sedentary behavior is not associated with the risk of binge drinking (see Table 6).

**Table 6:** Logistic regression analysis results for sedentary behavior and binge drinking.

Mode	*b*	SE	Wald	*P*	OR (95%CI)
VII	−0.123	0.071	2.988	0.084	0.884 (0.770 ~ 1.017)
VII	0.049	0.077	0.411	0.521	1.051 (0.904 ~ 1.221)

VII: Original model without adjustment for any variables. VII: Adjustment all covariates.

### 3.2 Study 2: Daily tracking survey

**Figure 5:**
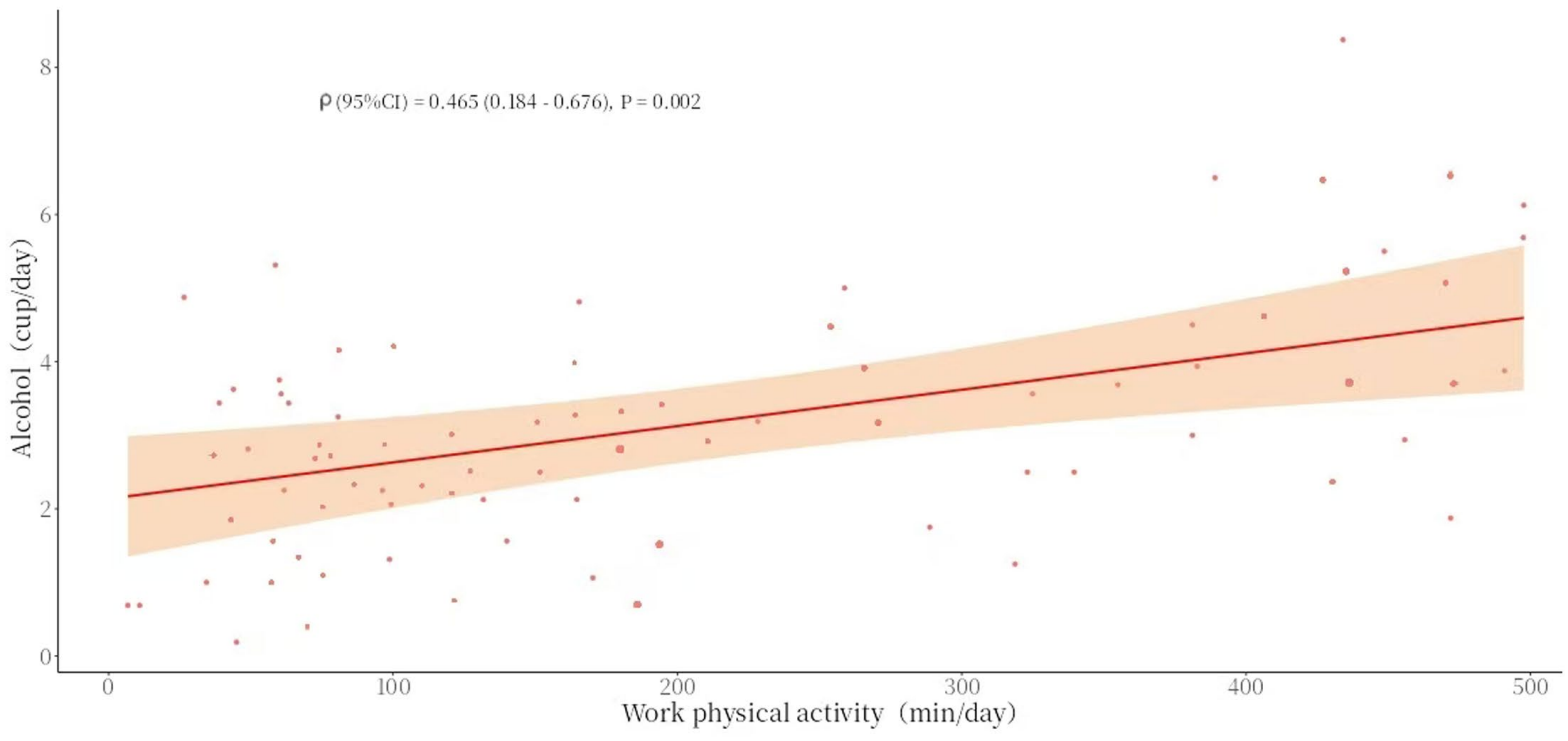
Scatter chart of work physical activity (mean) and alcohol consumption (mean).

**Figure 6:**
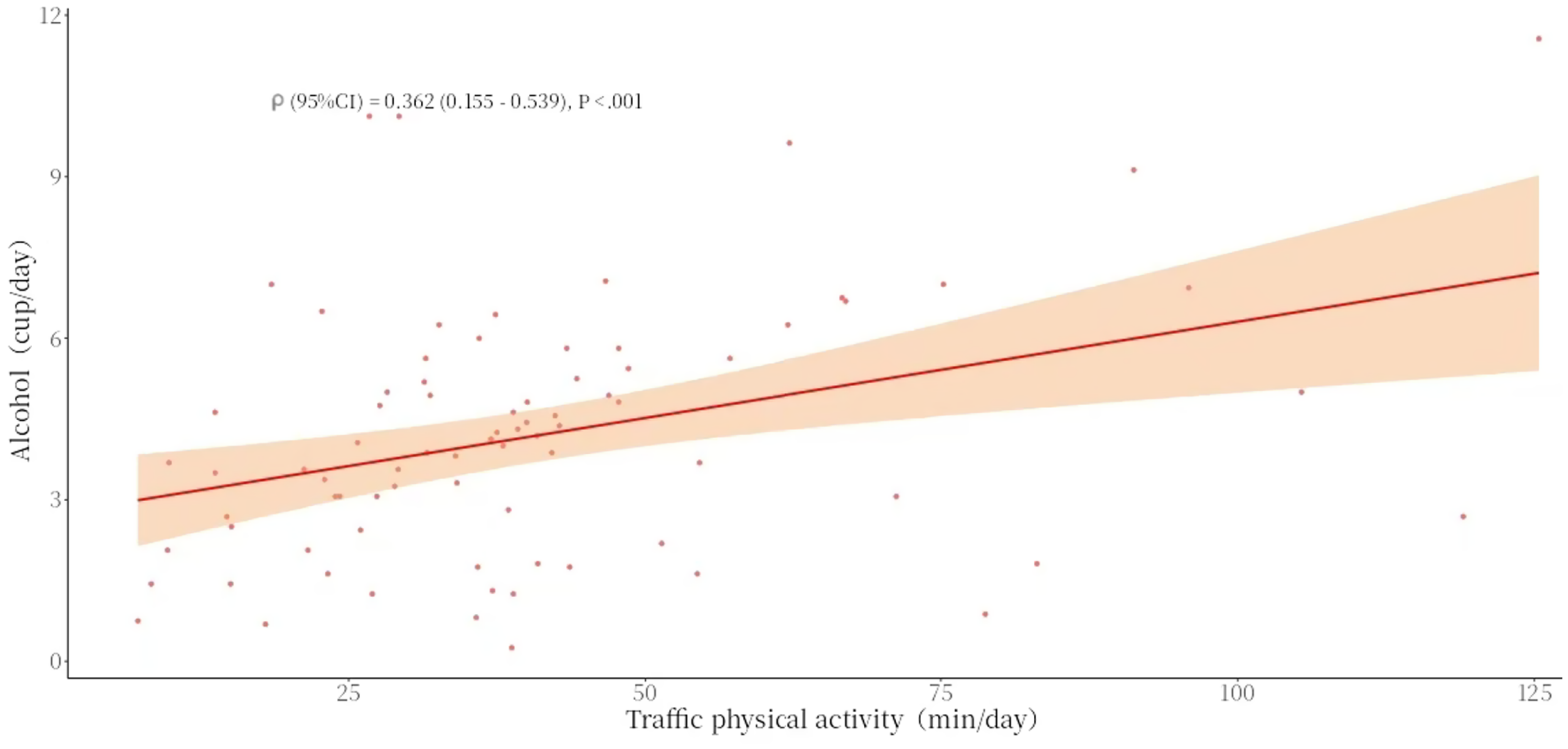
Scatter chart of traffic physical activity (mean) and alcohol consumption (mean).

Spearman’s correlation analysis showed that there was a statistically significant association between the average daily duration of work-related physical activity and average daily alcohol consumption (*ρ* = 0.465, *p* = 0.002), and similarly, there was a statistically significant association between the average daily duration of transportation-related physical activity and average daily alcohol consumption (*ρ* = 0.362, *p* < 0.001) (see Table 7 and Figures 5, 6).

**Table 7:** Correlation analysis between work physical activity, transportation physical activity and alcohol consumption (mean).

Variable	*N*	*ρ*	*P*
Average work physical activity * average alcohol consumption	80	0.465	0.002**
Average transportation physical activity * average alcohol consumption	80	0.362	<0.001***

*p < 0.05, **p < 0.01, ***p < 0.001.

The results of the generalized linear mixed model (negative binomial regression) analysis showed that the absolute value of the *O*-value was greater than 1.96, negative binomial regression should be used for testing. Model 1: Without controlling for demographic variables, each additional minute of work physical activity was associated with an average increase of 0.002 cups in alcohol consumption. Work physical activity duration is positively correlated with alcohol consumption (*p* < 0.001, 95% CI: 0.002–0.008). In Model 2, after adjust all covariates (age, gender, education level, marital status, BMI, and health status), each additional minute of work physical activity duration is associated with an average increase of 0.001 cups in alcohol consumption. The duration of work physical activity remained positively correlated with alcohol consumption (*p* < 0.001, 95% CI: 0.001–0.003). The narrower CI (0.001–0.003) after adjusting for covariates reflects greater precision and stability (see Table 8).

**Table 8:** Negative binomial regression of work physical activity, transportation physical activity and binge drinking.

Model	*β*	SE	*t*	*Z*	*O*	*P*	95%CI
Model 1	0.002	0.0002	9.598	5.802	16.745	<0.001***	(0.002–0.008)
Model 2	0.001	0.0002	9.057	4.450	16.745	<0.001***	(0.001–0.003)
Model 3	0.011	0.001	15.676	8.811	24.642	<0.001***	(1.009–1.014)
Model 4	0.010	0.001	14.946	7.847	24.642	<0.001***	(1.008–1.013)

Model 1: Original model without adjustment for any variables. Model 2: adjusts for all covariates. Model 3: Original model without adjustment for any variables. Model 4 adjusts for all covariates. *p < 0.05, **p < 0.01, ***p < 0.001.

Model 3: Without controlling for demographic variables, each additional minute of transportation physical activity duration was associated with an average increase of 0.011 cups in alcohol consumption. Transportation physical activity duration is positively correlated with alcohol consumption (*p* < 0.001, 95% CI: 0.009–0.014). In Model 4, after adjust all covariates (age, gender, education level, marital status, BMI, and health status), each additional minute of transportation physical activity duration was associated with an average increase of 0.010 cups in alcohol consumption. The duration of transportation physical activity remained positively correlated with alcohol consumption (*p* < 0.001, 95% CI: 0.008–0.013). The narrower CI (0.008–0.013) after adjusting for covariates reflects greater precision and stability (see Table 8).

### 3.3 Study 3: randomized controlled trial

#### 3.3.1 Demographics

**Figure 7:**
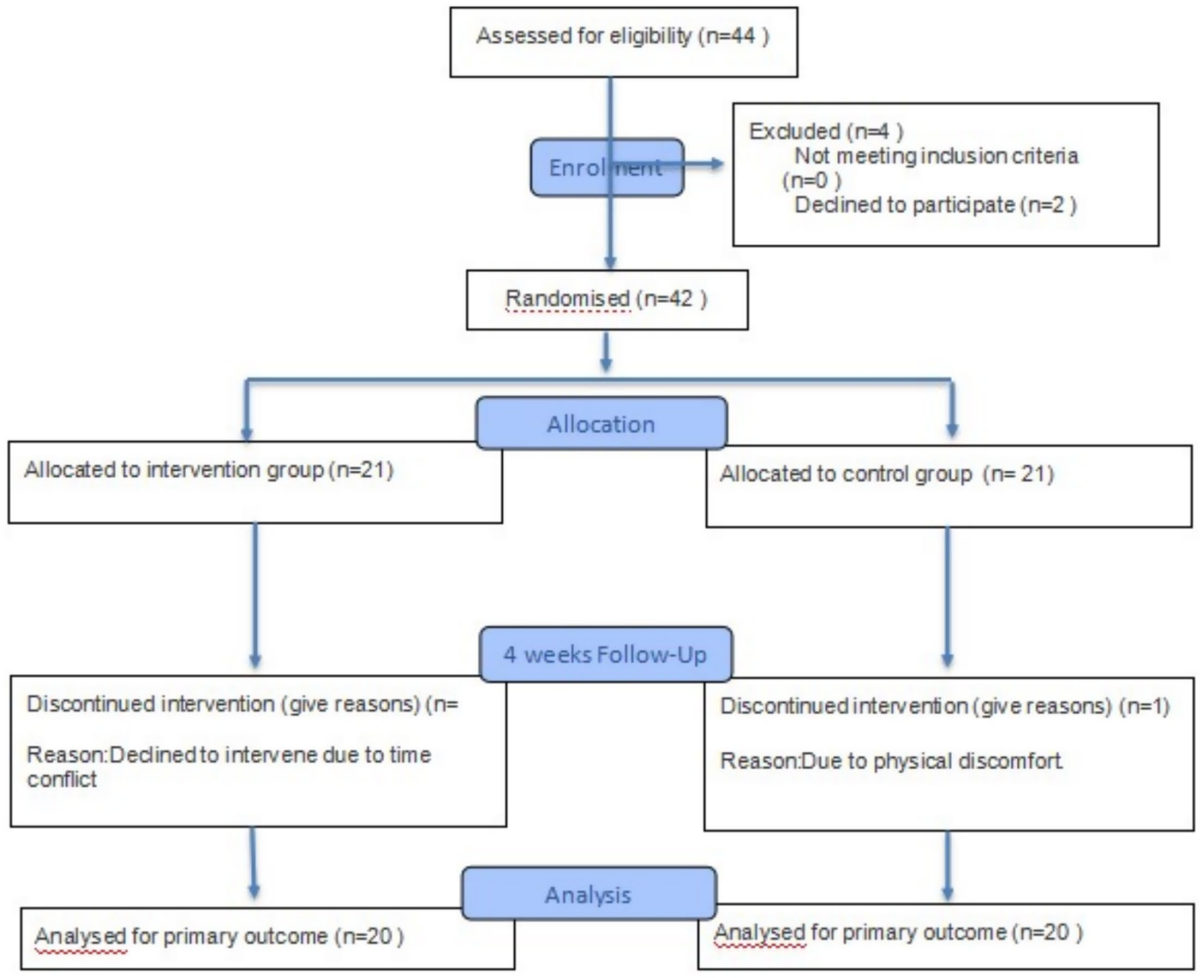
Participant flow chart.

This study surveyed a total of 40 subjects aged 39.4 ± 9.68 years, who provided demographic information data on age, gender, and BMI. Flowchart for recruiting participants (see Figure 7). There were no statistically significant differences between the control group and the experimental group in terms of age (*p* = 0.067) and BMI (*p* = 0.744) before the intervention.

#### 3.3.2 Relationship between recreational physical activity and binge drinking

The paired sample results showed that the mean value of the control group before and after the experiment was 0.75 with a standard deviation of 1.87, and the *p* > 0.05, indicating no significant difference. The mean value of the experimental group before and after the experiment was 4.45 with a standard deviation of 1.84, and the *p* < 0.001, indicating a significant difference (see Table 9).

**Table 9:** Intra-group differences in recreational physical activity and binge drinking.

Variable	*N*	M ± SD	*t*	*P*	95%CI
(Control group) experiments before and after	20	0.75 ± 1.87	0.18	0.86	(−0.80–0.95)
(Experimental group) experiments before and after	20	4.45 ± 1.84	10.81	<0.001***	(3.59–5.31)

*p < 0.05, **p < 0.01, ***p < 0.001.

Before intervention, an independent samples *t*-test was performed on the alcohol consumption levels of the control group (6.55 ± 1.81) and the experimental group (6.63 ± 2.30) (see Table 10). The *p* > 0.05, which did not reach statistical significance, indicating that the alcohol consumption levels of the two groups were homogeneous.

**Table 10:** Intergroup differences in recreational physical activity and binge drinking.

Variable	*N*	Control group	Experimental group	*P*
Binge drinking (Pre-test)	40	6.55 ± 1.81	6.63 ± 2.30	>0.05
Binge drinking (Post-test)	40	5.48 ± 2.80	2.18 ± 2.01	<0.001***

*p < 0.05, **p < 0.01, ***p < 0.001.

After the intervention, there was a significant difference between the control group (5.48 ± 2.80) and the experimental group (2.18 ± 2.01) in terms of recreational physical activity and alcohol consumption, with a *p* < 0.001. The data indicate that, over time, the experimental group performed better than the control group in reducing alcohol consumption through recreational physical activity intervention (see Table 10).

**Figure 8:**
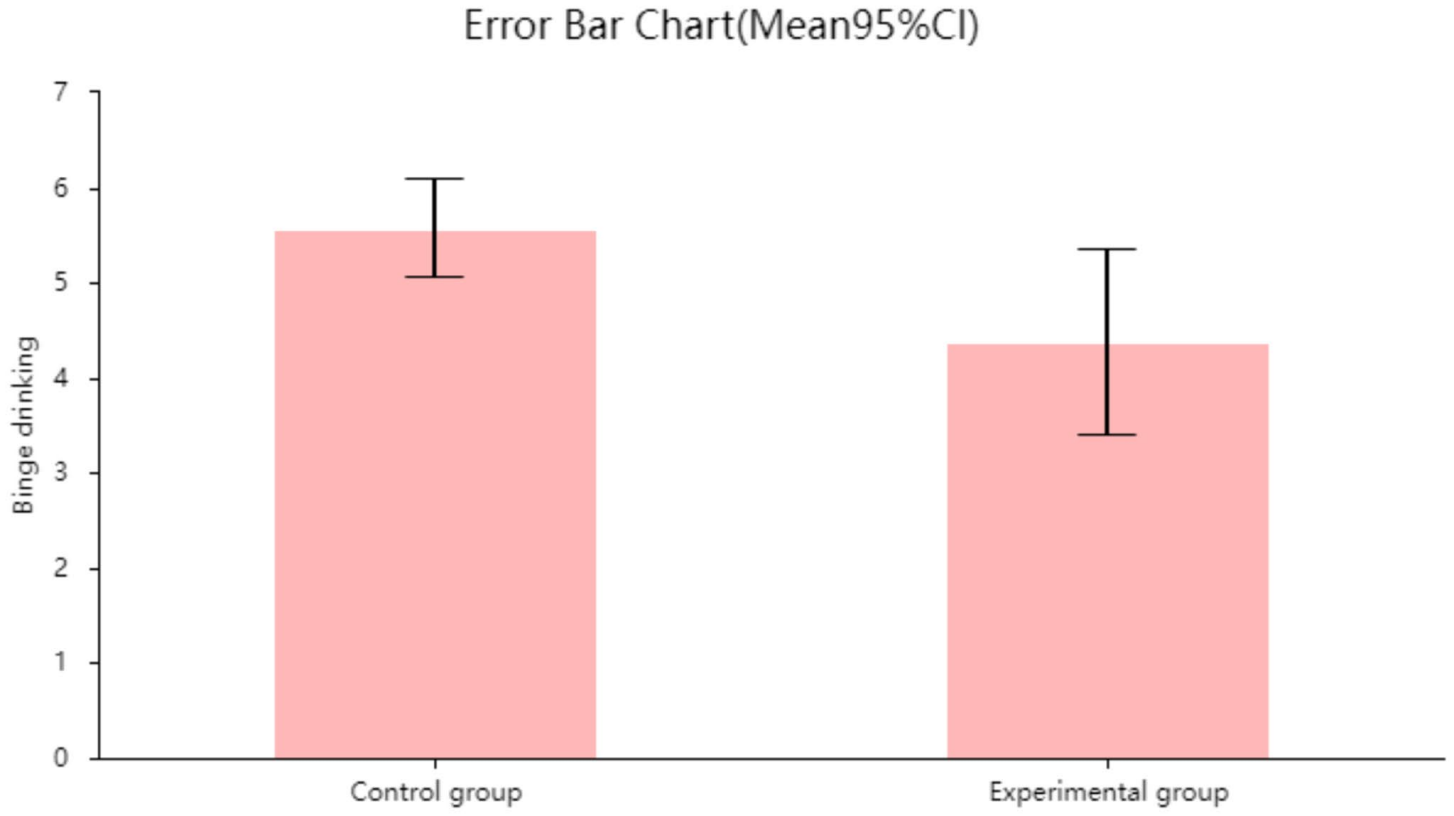
Error chart of the relationship between recreational physical activity and binge drinking in the control group and the experimental group.

As shown in the error chart results, the mean alcohol abuse score in the experimental group (mean = 4.348, 95% CI [3.404–5.356]) was significantly lower than that in the control group (mean = 5.567, 95% CI [5.052–6.082]). Since the 95% confidence intervals for the means of the two groups do not overlap at all, this indicates that the difference between the experimental and control groups is highly statistically significant (*p* < 0.001), and the experimental intervention significantly associated the severity of alcohol abuse (see Figure 8).

## 4 Discussion

### 4.1 Work physical activity and binge drinking

Cross-sectional survey results and daily tracking survey results are consistent, they indicating that the longer the duration of work-related physical activity, the higher the probability of alcohol abuse risk. In cross-sectional results, the risk of alcohol abuse associated with work physical activity is higher than that associated with transportation physical activity. However, in daily follow-up surveys, this result is reversed, with the increase in alcohol abuse risk from work physical activity being slightly lower than that from transportation physical activity. This discrepancy may be attributed to cultural, environmental, and institutional factors. In China, individuals with longer durations of work physical activity tend to have lower incomes and heavier family burdens, making it difficult to afford excessive alcohol consumption (42). In contrast, those with longer durations of transportation physical activity may have more time and financial resources available for drinking.

Past research indicates that individuals engaged in work physical activity are more likely to engage in binge drinking than those not involved in such activities, with the duration of work physical activity increasing the risk of developing an addiction to heavy drinking (19, 38). This may be based on the following reasons: Participants engaged in moderate to vigorous work physical activity experience greater psychological and physiological stress, leading to an increased likelihood of binge drinking, which is viewed as a form of ‘self-medication’ (43). The longer the duration of work physical labor, the fewer opportunities for sleep, and drinking becomes a compensatory strategy (44). The threshold effect shows that when work physical activity duration is less than 445 min, there is a positive correlation between work physical activity and the risk of binge drinking. Approximately 445 min equals 7.42 h. After work, individuals have more free time, and influenced by social factors, more people choose socializing and dining culture as one of the ways to relax and build relationships with peers (45). It is worth noting that frequently using alcohol to combat fatigue can reinforce the habit through the reward mechanism (dopamine pathways) (46).

### 4.2 Transportation physical activity and binge drinking

The results of cross-sectional surveys and daily tracking surveys are broadly consistent, indicating that (1) transportation physical activity and the probability of binge drinking are also positively correlated. People who engage in physical activity related to transportation are 31% more likely to abuse alcohol than those who do not. (2) The longer the duration of transportation physical activity, the higher the risk of excessive alcohol consumption.

Related studies indicate that prolonged transportation physical activity (such as delivery work, long-distance walking, or cycling), high psychological stress, and a sense of urgency lead to significant physical fatigue, soreness, stress, and sleep disorders, thereby increasing the tendency toward stress-related binge drinking (47), with alcohol becoming a tool to cope with emotions. On the other hand, the unstable income associated with transportation physical activity exacerbates the risk of using alcohol to escape negative emotions (48). Transportation physical activities primarily occur during commuting hours (7:00 a.m.–9:00 a.m.; 4:00 p.m.–7:00 p.m.), but post-work transportation physical activities typically last longer (49). As the day’s work and exercise conclude, the body and mind fully relax, and more people are inclined to use alcohol or alcoholic beverages to alleviate fatigue. This phenomenon is referred to as the “time-relaxation window and binge drinking risk” in occupational health research (44). Research has found that the effect of drinking after work is significant, with incidence rates peaking 2–4 h after work (48).

However, there exists a slight discrepancy between daily tracking surveys and cross-sectional studies. In our daily tracking survey, we did not observe a positive correlation between transportation physical activity and alcohol abuse within the specific timeframe (35–145 min) examined in the cross-sectional study. The inconsistency in this finding may be attributed to the smaller sample size of the daily tracking population and the regional characteristics of transportation modes. Chinese people’s travel modes are influenced by the pressures of study, life, and work, with most opting for public transport, private cars, electric scooters, and motorcycles as means of transportation. Different individuals may choose travel modes based on their specific circumstances, which could weaken the association between the duration of physical activity during commuting and alcohol abuse. Therefore, this may also be a major factor in the absence of a specific time range.

### 4.3 Recreational physical activity and binge drinking

Individuals who regularly participate in recreational sports activities have a lower probability of binge drinking (50). The regression model in the cross-sectional survey indicates that individuals who engage in moderate to vigorous recreational physical activity have a lower probability of binge drinking than those who do not participate in recreational physical activity, with a 40–50% reduction in the probability of binge drinking risk (50).

The randomized controlled trial results and error bar charts show that group-based recreational physical activities significantly reduce alcohol consumption. The distribution of daily drinking time and alcohol intake per individual indicates that the evening accounts for the largest proportion of daily alcohol intake. As the duration of recreational physical activity interventions increases, both alcohol intake and drinking frequency decrease, and the evening proportion gradually decreases. The extent of the decrease varies from person to person, and a plateau may occur after 1–2 consecutive days of decline. Participants reported that individuals with relatively lower alcohol consumption were more likely to control their daily alcohol intake and evening drinking tendencies. Most participants indicated that engaging in group exercise in the evening and avoiding evening social gatherings were important strategies for controlling evening drinking. However, a small number of participants (those with higher alcohol consumption) reported feeling a strong desire to drink the morning after exercising.

Related studies indicate that voluntarily and actively engaging in recreational physical activities can effectively reduce negative emotions such as stress, anxiety, and depression (51). This is primarily because recreational physical activity inherently focuses on pleasure, engagement, and relaxation. The combination of physical stimulation and mental pleasure activates the cerebral cortex, stimulating the release of hormones such as endorphins, thereby influencing mood and improving unhealthy lifestyles (52). And coordinated and sustained recreational physical activities can enhance prefrontal cortex function, improving decision-making ability and impulse control. This region is a deficit area in high-risk populations for binge drinking (53). By enhancing prefrontal cortex plasticity through exercise, self-control can be improved, reducing the occurrence of binge drinking. Individuals who choose recreational physical activities replace the time and settings associated with binge drinking, opting for activities like dancing or basketball after work instead of social drinking at gatherings (54). The pursuit of physical appearance and aesthetic appeal may also lead to reduced alcohol consumption. In particular, activities such as dancing, yoga, Pilates, and aerobics place a strong emphasis on physical appearance, and the negative effects of alcohol on body shape, skin, and mental state may prompt individuals to exercise self-restraint.

### 4.4 Sedentary behavior and binge drinking

There is no obvious association between sedentary behavior and binge drinking. The results from the chi-square test in the cross-sectional study indicate that there is no significant difference between the binge drinking group and the non-binge drinking group in terms of sedentary behavior. This is inconsistent with the conclusions of previous studies (20, 32) and is worthy of discussion. Previous studies on health behaviors during the 2019 COVID-19 pandemic (HEBECO) found an association between sedentary behavior and binge drinking among British adults (20). The pandemic may explain this phenomenon, as adults were only permitted to exercise indoors, disrupting many people’s habits regarding fitness venues and exercise schedules. Familiar indoor or home environments may have made it easier for individuals to abandon exercise, opting instead for more comfortable lifestyle choices (20). However, current literature on the relationship between sedentary behavior and excessive alcohol consumption is inconsistent. The ‘behavioral clustering theory’ suggests that sedentary behavior often co-occurs with other unhealthy lifestyle choices (high-calorie diets, smoking, and drinking), as these are driven by the same psychological motivations (boredom, stress) (45). Additionally, sedentary behavior is closely linked to depression and anxiety, with some individuals turning to alcohol to cope with negative emotions (55).

### 4.5 Limitations of this study

This study has the following limitations: (1) incomplete adjustment for covariates; alcohol abuse has multiple causes, including genetic, behavioral, environmental, and social factors. Since these factors were not included in the NHANES database, this study was unable to exclude all confounding factors. (2) In this study, work-related physical activity and transportation-related physical activity were assessed based on self-reporting, despite external validation. Self-reporting is inherently subjective and may introduce bias into the study results.

In future studies, we will strive to avoid the aforementioned limitations and further refine the classification of recreational physical activities based on the innovative aspects of this study, aiming to identify activity factors that can reduce the risk of binge drinking. If the classification of physical activities is more aligned with real-world scenarios, linking recreational physical activity patterns to binge drinking may hold greater clinical significance.

## 5 Conclusion

Daily work physical activity and transportation physical activity were positively associated with binge drinking, while daily recreational physical activity was negatively associated with binge drinking. There was no significant association between sedentary behavior and binge drinking. In a representative sample of Chinese adults, we also confirmed results consistent with those from a U.S. adult sample, namely a negative association between transportation physical activity and binge drinking, and a causal relationship between recreational physical activity and binge drinking. Therefore, not all physical activity reduces the likelihood of binge drinking, and the types of physical activity can have both positive and negative effects on binge drinking. Thus, selecting moderate recreational physical activity to avoid binge drinking is an appropriate approach.

## Data Availability

The original contributions presented in the study are included in the article/supplementary material, further inquiries can be directed to the corresponding author.

## Footnotes

1
https://www.cdc.gov/nchs/nhanes/index.htm

## Glossary

SPSS: Statistical Product and Service Solutions
p: *p*-value
COVID-19: Corona Virus Disease 2019
CAPI: Computer-Assisted Personal Interviewing
NHANES: National Health and Nutritional Examination Survey
BMI: body mass index
OR: Odds Ratio
GPAQ: Global Physical Activity Questionnaire
CI: Confidence Interval
LLCI: Lower limit confidence interval
ULCI: Upper limit confidence interval
SE: Standard error
RCS: Restricted Cubic Spline
WHO: World Health Organization
NIAAA: National Institute on Binge Drinking and Alcoholism
CDC: Centers for Disease Control and Prevention
N: Sample size
*ρ*: Correlation coefficient
*β*: regression coefficient
t: *t*-statistic
Z: Standard normal deviate
O: Passed the dispersion test
F: Joint hypotheses test
M: Mean
SD: Standard deviation
